# The Transfer of the Hepatocyte Growth Factor Gene by Macrophages Ameliorates the Progression of Peritoneal Fibrosis in Mice

**DOI:** 10.3390/ijms24086951

**Published:** 2023-04-09

**Authors:** Yoko Obata, Katsushige Abe, Masanobu Miyazaki, Takehiko Koji, Yasuhiko Tabata, Tomoya Nishino

**Affiliations:** 1 Department of Nephrology, Nagasaki University Graduate School of Biomedical Sciences, 1-7-1 Sakamoto, Nagasaki 852-8501, Japan; tnishino@nagasaki-u.ac.jp; 2 Abe Diabetes Clinic, 16-13 Nakakasuga-machi, Oita 870-0039, Japan; rukonyan@ga2.so-net.ne.jp; 3 Miyazaki Clinic, 3-12 Shiratori-cho, Nagasaki 852-8042, Japan; msnbmiya@gmail.com; 4 Department of Histology and Cell Biology, Nagasaki University Graduate School of Biomedical Sciences, 1-12-4 Sakamoto, Nagasaki 852-8523, Japan; 5 Laboratory of Biomaterials, Department of Regeneration Science and Engineering, Institute for Life and Medical Sciences, Kyoto University, 53 Kawara-cho Shogoin, Sakyo-ku, Kyoto 606-8507, Japan; yasuhiko@infront.kyoto-u.ac.jp

**Keywords:** peritoneal dialysis, peritoneal fibrosis, macrophage, hepatocyte growth factor, cationized gelatin microspheres

## Abstract

Growing evidence indicates that hepatocyte growth factor (HGF) possesses potent antifibrotic activity. Furthermore, macrophages migrate to inflamed sites and have been linked to the progression of fibrosis. In this study, we utilized macrophages as vehicles to express and deliver the HGF gene and investigated whether macrophages carrying the HGF expression vector (HGF-M) could suppress peritoneal fibrosis development in mice. We obtained macrophages from the peritoneal cavity of mice stimulated with 3% thioglycollate and used cationized gelatin microspheres (CGMs) to produce HGF expression vector-gelatin complexes. Macrophages phagocytosed these CGMs, and gene transfer into macrophages was confirmed in vitro. Peritoneal fibrosis was induced by intraperitoneal injection of chlorhexidine gluconate (CG) for three weeks; seven days after the first CG injection, HGF-M was administered intravenously. Transplantation of HGF-M significantly suppressed submesothelial thickening and reduced type III collagen expression. Moreover, in the HGF-M-treated group, the number of α-smooth muscle actin- and TGF-β-positive cells were significantly lower in the peritoneum, and ultrafiltration was preserved. Our results indicated that the transplantation of HGF-M prevented the progression of peritoneal fibrosis and indicated that this novel gene therapy using macrophages may have potential for treating peritoneal fibrosis.

## 1. Introduction

Peritoneal dialysis (PD) is an established renal replacement therapy for patients with end-stage renal disease. However, it is widely known that long-term PD therapy leads to various structural changes in the peritoneal membrane, characterized by the loss of mesothelial cells, marked peritoneal fibrosis, and massive accumulation of collagen in the peritoneum [1,2,3]. Furthermore, these structural changes in the peritoneum induce alterations in the peritoneal function, including reduced ultrafiltration and increased small-solute transport rates [4]. These changes in the peritoneum often hinder PD continuation. However, the molecular mechanisms involved in the initiation and progression of peritoneal fibrosis remain unclear, and effective prevention and treatment strategies have not been established.

Hepatocyte growth factor (HGF) is widely known to be a multifunctional growth factor that was originally identified as a potent mitogen for hepatocytes [5]. It reportedly exerts several functions, including anti-apoptotic [6], anti-inflammatory [7], and anti-fibrotic [8] abilities. Several studies have reported the therapeutic significance of HGF in injury/disease models including acute tissue injury [9,10], chronic fibrosis [11,12,13], and cardiovascular diseases [14,15]. Thus, we hypothesized that HGF may be a target for peritoneal fibrosis treatment. 

Recently, the progress of gene therapy has been remarkable, and clinical application has begun for hereditary diseases [16,17,18,19,20], malignant tumors [21,22], neurodegenerative diseases [23,24], and cardiovascular diseases [25,26]. Several viral and non-viral vectors have been explored to enhance gene transfection efficiency. Although non-viral vectors have several advantages over viral vectors, including low toxicity and immune responses, they may be degraded by nucleolytic enzymes in vivo before they are introduced into cells; therefore, introduction efficiency remains a limitation. Previously, we utilized cationized gelatin microspheres (CGMs) as non-viral vectors to protect DNA against rapid degradation and to allow the sustained release of small interfering RNA (siRNA) [27,28]. Gelatin has been extensively used in industrial, pharmaceutical, and medical applications, and its biosafety has been proven through its clinical use as a surgical biomaterial and drug ingredient [29]. Another advantage of gelatin is that it can be freely shaped and sized. Furthermore, the degradation profile of cationized gelatin hydrogels can be changed by altering the concentration of glutaraldehyde used to crosslink cationized gelatin [30]. 

We previously reported that macrophage infiltration is associated with peritoneal fibrosis and the deterioration of peritoneal function [31,32]. Moreover, peritoneal macrophages in the fibrotic peritoneum are considered the major source of inflammatory and pro-fibrotic cytokines, such as transforming growth factor-beta (TGF-β). Typically, macrophages possess phagocytic capacity and can migrate to inflamed sites. Thus, in this study, we focused on these macrophage characteristics and aimed to utilize macrophages as a vehicle for expressing and delivering *HGF* gene at the peritoneum by ingesting CGMs that contained HGF-expressing plasmids. 

We investigated whether HGF, an anti-fibrotic factor, can prevent the progression of peritoneal fibrosis by using macrophages to establish an efficient *HGF* gene delivery.

## 2. Results

### 2.1. Confirmation of Gene Expression in the Macrophages In Vitro and In Vivo

**Figure 1:**
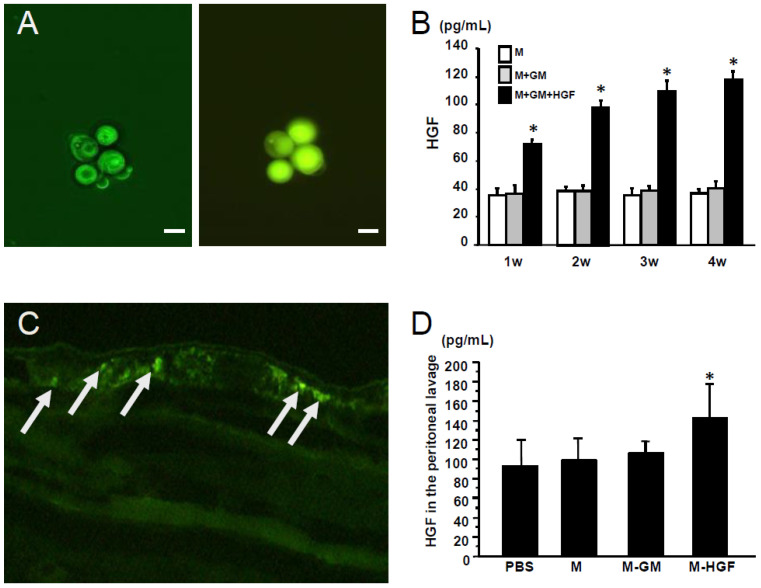
Confirmation of gene expression in the macrophages. (**A**) Left panel; macrophages phagocyte cationized gelatin microspheres (CGMs) incorporating green fluorescent protein (GFP) expression plasmid by light microscope. Right panel; in macrophages phagocyted CGMs, GFP expression was detected by fluorescence microscope. Scale bar; 100 mm, magnification; 40×; (**B**) Hepatocyte growth factor (HGF) levels in the culture medium. M; macrophages only, M + GM; macrophages and gelatin microspheres, M + GM + HGF; macrophages and gelatin microspheres incorporating *HGF* gene. * denotes a significant difference against the M group at *p* < 0.05. (**C**) Arrows show GFP expressing macrophages in the chlorhexidine gluconate (CG) induced fibrotic peritoneum at 1 week after transplantation. Magnification; 200×; (**D**) HGF levels in the peritoneal lavage fluid. Phosphate-buffered solution (PBS); CG injected mice administered PBS-CGMs, M; CG injected mice administered macrophages, M-GM; CG injected mice administered macrophages phagocyting CGMs, M-HGF; CG injected mice administered macrophages phagocyting CGMs incorporating *HGF* gene. * denotes a significant difference against the PBS group at *p* < 0.05.

First, we confirmed the incorporation of the CGM-*green fluorescent protein (GFP)* plasmid complexes and GFP expression in macrophages in vitro (Figure 1A). Moreover, an increase in HGF levels in the culture medium of M-HGF was observed after 4 weeks (Figure 1B). Next, we examined the localization of macrophages transfected with the *GFP* expression plasmid using CGMs in the CG-induced peritoneal fibrosis model. We confirmed the presence of GFP-positive cells in the submesothelial compact zone in the peritoneum (Figure 1C). According to the examination of HGF levels by enzyme-linked immunosorbent assay (ELISA) in peritoneal lavage fluid, HGF levels significantly increased in mice injected with macrophages phagocytosed with CGM-*HGF* gene complexes (Figure 1D). 

### 2.2. M-HGF Attenuates Peritoneal Fibrotic Thickening Induced by CG

**Figure 2:**
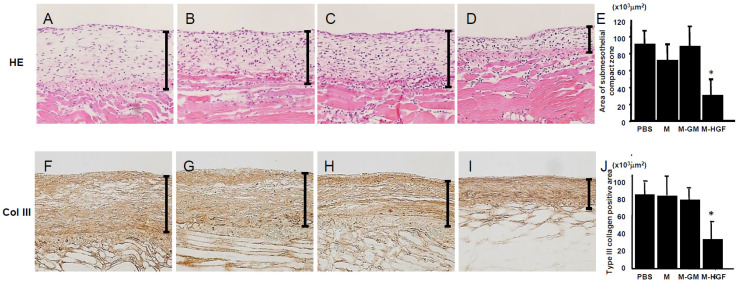
HE staining (upper panel) and immunohistochemistry findings for type III collagen (lower panel) of peritoneal tissues at day 21. In the PBS group (**A**), CG induced marked thickening of peritoneal tissue. In the M group (**B**) and M-GM group (**C**), marked thickening of the submesothelial compact zone was observed, similar to that found in the PBS group at 21 days. In the M-HGF group (**D**), progression of peritoneal thickening was suppressed at 21 days. (**E**) The bar graph shows the results of the semi-quantitative analyses of the submesothelial compact zone. In the PBS group (**F**), the expression of type III collagen markedly increased in the thickened submesothelial compact zone at 21 days. In the M (**G**) and M-GM (**H**) groups, the expression levels of type III collagen were greatly increased in the submesothelial compact zone at 21 days, similar to the PBS group. In the M-HGF group (**I**), type III collagen expression decreased in the submesothelial compact zone at 21 days. (**J**) The bar graph shows the results of the semi-quantitative analyses of type III collagen-positive areas. * denotes a significant difference compared with the PBS group at *p* < 0.05. Bars indicate the thickness of the submesothelial compact zone; magnification, 200×.

After hematoxylin and eosin (HE) staining on day 21, peritoneal tissues thickened, and we observed fibrosis of the peritoneal tissues in the phosphate-buffered solution (PBS) group (Figure 2A). The thickness of the submesothelial compact zone in the M and M-GM groups was the same as that in the PBS group (Figure 2B,C). In contrast, administration of macrophages carrying HGF (M-HGF group) significantly prevented submesothelial thickening in CG-injected mice (Figure 2D). Furthermore, we conducted immunohistochemistry for type III collagen, which is a major component of the submesothelial thickening. In the PBS group, type III collagen was diffusely expressed in the submesothelial compact zone (Figure 2F), whereas its expression was clearly lowered in the M-HGF group (Figure 2I). The positive areas for type III collagen in the M group (Figure 2G) and M-GM group (Figure 2H) were similar to those in the PBS group. Figure 2E,J shows the results of semi-quantitative analyses of the submesothelial compact zone and the type III collagen-positive area. We observed no influence on the body weight, and the liver and renal function in all groups by macrophage transplantation or *HGF* gene delivery (Table 1).

### 2.3. M-HGF Suppresses TGF-β Expression, Reduces the Number of Myofibroblasts and Suppresses Cell Proliferation

**Figure 3:**
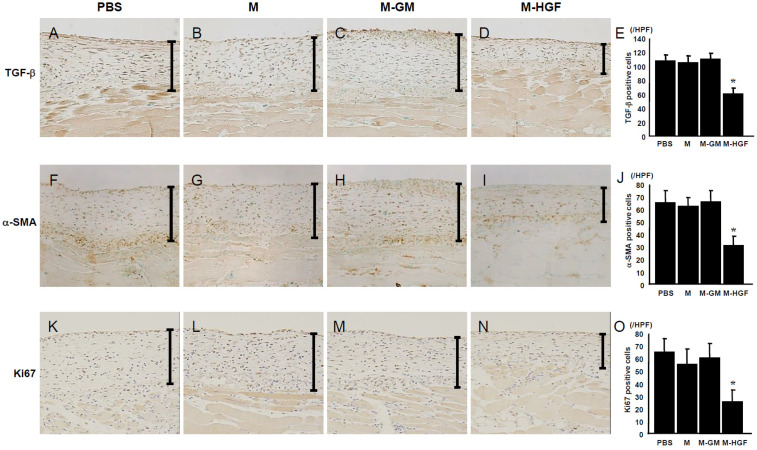
Immunohistochemistry findings for transforming growth factor-beta (TGF-β), alpha-smooth muscle actin (α-SMA), and Ki67. In the PBS group (**A**,**F**), the number of TGF-β-and αSMA-positive cells markedly increased in the thickened submesothelial compact zone at 21 days, while the proliferation of TGF-β-and α-SMA-positive cells in the submesothelial compact zone decreased in the M-HGF group at 21 days (**D**,**I**). In the M group (**B**,**G**) and M-GM group (**C**,**H**), the number of TGF-β-and α-SMA-positive cells markedly increased in the thickened submesothelial compact zone at 21 days, similar to that in the PBS group. The bar graph shows the number of TGF-β-and α-SMA-positive cells (**E**,**J**). In addition, the number of Ki67-positive cells significantly decreased in the fibrotic peritoneum of the M-HGF group (**N**) compared with those of the PBS (**K**), M (**L**), and M-GM (**M**) groups. The bar graph shows the number of Ki67-positive cells (**O**). * denotes a significant difference compared with the PBS group at *p* < 0.05. The bars indicate the thickness of the submesothelial compact zone; magnification, 200×.

Since TGF-β is an important factor that mediates the development of fibrosis [33] and counteracts the action of HGF [34,35], we evaluated the number of TGF-β-positive cells in the submesothelial compact zone. Twenty-one days after CG injection, many TGF-β-positive cells were detected in the thickened peritoneal tissue in the PBS group (Figure 3A). Similarly, TGF-β-positive cells were increased in the thickened peritoneum in the M group (Figure 3B) and M-GM group (Figure 3C). However, a significantly lower number of TGF-β-positive cells were detected in the M-HGF group (Figure 3D,E). Furthermore, we performed immunohistochemistry for alpha-smooth muscle actin (α-SMA), a myofibroblast marker that plays an important role in the progression of fibrosis. In the PBS group on day 21, many α-SMA -positive cells were observed in the thickened submesothelial compact zone (Figure 3F). Compared with the PBS group, fewer α-SMA-positive myofibroblasts were found in the M-HGF group (Figure 3I,J), whereas the number of myofibroblasts in the M and M-GM groups was similar to that in the PBS group (Figure 3G,H). Next, we performed the immunohistochemistry for Ki67 to assess the cell proliferation in the fibrotic peritoneum. As a result, the number of Ki67-positive cells was significantly decreased in the peritoneum of the M-HGF mice compared with those in the PBS, M, and M-GM groups (Figure 3K–O).

### 2.4. M-HGF Attenuates the Peritoneal Hyperpermeability

**Figure 4:**
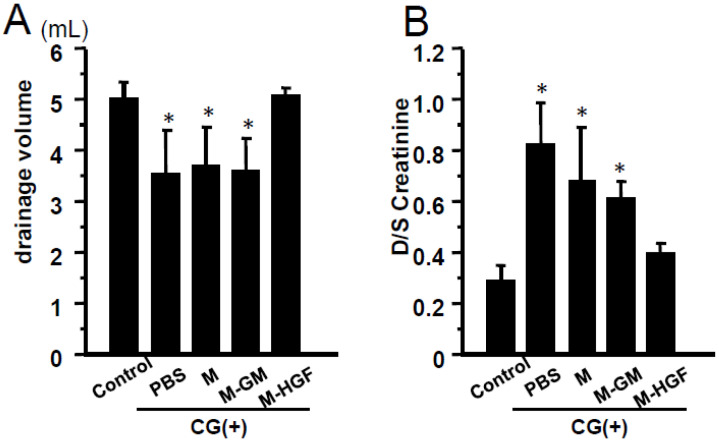
The results of peritoneal equilibration test. (**A**) Bar graph showing dialysate drainage fluid volume. The result exhibited lower drained volume in the PBS, M, and M-GM groups compared with the control group. Drained volume in the M-HGF group was maintained and was significantly increased compared to the PBS group. (**B**) The bar graph showing Dialysate (D)/Serum (S) ratio of creatinine. The result showed a higher D/S ratio of creatinine in the PBS, M, and M-GM groups compared with the control group. The D/S ratio of creatinine in the M-HGF group was the same as that in the control group and it was lower compared to the PBS group. * denotes a significant difference against the control group at *p* < 0.05.

The peritoneal function was examined using a modified peritoneal equilibration test (PET) system. In CG-injected mice, significant impairment of ultrafiltration was observed in the PBS, M, and M-GM groups compared to the mice not injected with CG; however, transplantation of M-HGF reversed the ultrafiltration dysfunction (Figure 4A). A significantly higher dilute/serum ratio of creatinine (D/S Cr) was observed in the CG-injected PBS, M, and M-GM groups than in the control group, suggesting a higher peritoneal permeability in the CG-injected mice. Meanwhile, the D/S Cr in the M-HGF group was the same as that in mice not injected with CG (Figure 4B).

## 3. Discussion

Our results showed that CGMs allowed efficient gene transfer into macrophages, indicating a novel cell-specific gene delivery system. Through an in vivo study, we confirmed that injecting M-HGF suppressed the expression of TGF-β, α-SMA, and type III collagen, consequently suppressing the progression of peritoneal fibrosis while maintaining peritoneal function.

Plautz et al. published the first report on gene transfer into specific cells in 1991 [36]. Since then, gene transfer using smooth muscle cells or endothelial cells has been conducted [37,38]. Inside cells, gene degradation by nucleic acid-degrading enzymes is suppressed, and gene expression is possible through gene transcription. Thus, specific cells can be administered in vivo by introducing genes into them ex vivo. The advantages of cell-based gene therapy are as follows: (1) selective gene transfer allows systemic administration, (2) genetically uniform cell populations can be developed, and (3) gene introduction and expression are assured. On the contrary, the disadvantages are as follows: (1) time is required for expression, (2) processes such as cell collection and culture are required, and (3) cell phenotype may change during culturing. Gene transfer into endothelial cells is conducted, as is coating of these endothelial cells into grafts [39] or stents [40]. However, its function as a place for gene expression has been emphasized in treatment, and treatment utilizing the characteristics of the cell itself has hardly been performed until now. Therefore, in this study, we focused on the phagocytic and migratory capacities of macrophages and utilized them as vectors for gene transfer. Tabata et al. reported that phagocytosis of foreign particles by macrophages is greatly affected by the properties of the particles themselves [41]. They found that macrophages prefer to take up a particular particle size and that gelatin exhibits a strong phagocytosis-promoting effect in the presence of serum. To perform gene-cell hybrid therapy, we used gelatin microspheres to introduce genes into cells. By forming a complex of gelatin and a gene, degradation by a nucleolytic enzyme can be prevented, and the gene can be safely and efficiently introduced. In addition, we utilized the properties of macrophages that specifically gather at the inflamed site and accomplished efficient *HGF* gene delivery to the peritoneum in this study. Similarly, Nagaya et al. reported a favorable therapeutic effect in a rat pulmonary hypertension model by introducing adrenomedullin into vascular endothelial progenitor cells with phagocytic capacity using gelatin particles [42]. Thus, not only macrophages but also cells with phagocytic capacity can be used as vectors for gene transfer. However, we believed that more efficient gene therapy could be achieved in this study by combining the properties of macrophages that accumulate at the site of inflammation and CGMs. In the future, it may be possible to isolate macrophages using an apheresis system. Then, ex vivo gene transfer using CGMs into isolated macrophages may lead to clinical application if adequate safety can be ensured and if they can be intravenously administered into the body.

Our study has some limitations. First, we did not examine the effect of the administration of macrophages or CGMs on the whole body in detail. However, we observed no influence on the survival rate and body weight, and no side effects on the liver and renal function in all groups (Table 1). In addition, GFP-positive macrophages were not observed in the liver or kidney. Second, we examined the rate of gene transfer into macrophages using CGMs. Although we observed GFP expression in about half of the macrophages by visual examination, detailed examination of the gene transfer efficiency might be needed using an objective method in the future. Third, we did not examine the dynamics of macrophages transfected HGF gene. At first, we tried to construct GFP-HGF plasmid or flag-HGF plasmid to confirm the location of macrophages transfected HGF gene, but we could not construct them successfully. Thus, we could only examine whether macrophages transfected with the GFP expression plasmid using CGMs could migrate to the CG-induced fibrotic peritoneum at day 14. In the future, it is desired to confirm the dynamics of macrophages by introducing the target gene at different times. Finally, we did not examine the effect of forcibly expressing HGF in macrophages on macrophage phenotype. Since HGF is multifunctional in regulating many cellular processes, it is possible to affect their phenotype. Based on the experimental results, we considered there was little effect on proliferation and apoptosis because there was no difference in the number of days to cell passage in vitro. Furthermore, M-HGF was slightly better in survival rate using trypan blue staining during passage, but there was no significant difference compared with other groups. However, other cytokines other than HGFwere not measured. On the other hand, the number of Ki67-positive cells significantly decreased in the fibrotic peritoneum of the M-HGF mice compared with those in the PBS, M, and M-GM groups (Figure 3). Thus, it is considered that the effect of HGF may change depending on the environment. Further studies are necessary to confirm the pathophysiological effects on the macrophage phenotype for clinical application.

In conclusion, our results indicate that HGF gene transfer by macrophages using CGMs is a novel gene therapy that may have potential for the treatment of peritoneal fibrosis.

## 4. Material and Methods

### 4.1. Preparation of CGMs

We used a gelatin prepared from acid-processed pig skin (sample kindly supplied by Nitta Gelatin Inc., Osaka, Japan) with an isoelectric point of 9.0 (MW 100,000). The ethylenediamine was purchased from Wako Pure Chemical Ltd. (Osaka, Japan). 2,4,6-Trinitrobenzene sulfonic acid (TNBS), 1-ethyl-3-(3-dimethylaminopropyl) carbodiimide hydrochloride salt (EDC), and 25% (*w*/*w*) aqueous glutaraldehyde solution were purchased from Nacalai Tesque (Kyoto, Japan). The gelatin carboxyl groups were introduced amino groups to cationize [30,43,44]. Briefly, ethylenediamine and EDC to 250 mL of 100 mM PBS containing 5 g of gelatin were added to accomplish this chemical conversion. For gelatin, the molar ratio of ethylenediamine to carboxyl groups was 50. After mixing immediately and adding 5 M HCl solution, the pH of the solution was adjusted to 5.0. The reaction mixture was agitated at 37 °C for 18 h and then dialyzed in a cellulose tube against double-distilled water (DDW) for 48 h at room temperature (RT). The dialyzed solution was freeze-dried, and we obtained the cationized gelatin used in our experiments. The percentage of amino groups introduced into gelatin when determined using the conventional TNBS method [45] was 50.9 mole percent per carboxyl group of gelatin. By chemically cross-linking gelatin in a water-in-oil emulsion, CGMs were prepared. An aqueous solution of 10% cationized gelatin (10 mL) was preheated to 40 °C and put dropwise to 375 mL of olive oil preheated to 40 °C, and then the solution was stirred by the impeller at 420 rpm for 10 min to yield a water-in-oil emulsion. The emulsion temperature was lowered to 4 °C, followed by further stirring for 30 min to turn into a gel from the aqueous gelatin solution. An amount of 100 mL cold acetone was added to this emulsion and stirred for 10 min to obtain microspheres. The obtained microspheres were washed with cold acetone three times, then collected by centrifugation. The size of the microspheres was fractionated by sieves with apertures of 70 and 100 µm, and the average of the microspheres’ diameter was 75 μm. The microspheres were air-dried at 4 °C, and then these non-cross-linked gelatin microspheres (50 mg) were stirred in a 25 mL acetone/0.01 M HCl solution (7/3, *v*/*v*), containing 60 μL of 25% glutaraldehyde solution at 4 °C for 24 h to allow the cationized gelatin to cross-link. For blocking the residual aldehyde groups of unreacted glutaraldehyde, the microspheres were washed using a short centrifugation with DDW and then agitated in 100 mM glycine solution (25 mL) at RT for 1 h. The obtained microspheres were washed with DDW three times by centrifugation and freeze-dried. As previously reported, the CGMs used in this study were designed to degrade within 2–3 weeks [30].

### 4.2. Preparation of Peritoneal Macrophages

Thioglycolate-elicited macrophages were obtained from the peritoneal cavity of male C57BL6/6J mice as previously described [46]. Briefly, mice were intraperitoneally injected with 3 mL of 3% thioglycolate (BD Biosciences Japan, Tokyo, Japan). Four days later, the exudate cells in the peritoneal cavity were collected by washing with PBS. After washing with cell culture medium containing RPMI 1640 supplemented with 10% fetal calf serum (FCS), 100 µg/mL penicillin, and 50 µg/mL streptomycin, the cells were seeded in a cell culture plate. The cells were then incubated at 37 °C overnight and washed with PBS to remove non-adherent cells. We used adherent monolayer cells as peritoneal macrophages.

### 4.3. Preparation of CGMs Incorporating Plasmids

We used pUC-SRα/HGF, kindly gifted by Dr. T Nakamura [5], and pEGFP-N3 (Takara Bio Inc., Shiga, Japan) as the DNA expression vector. To impregnate CGMs with either pUC-SRα/HGF or pEGFP-N3, PBS solution containing the plasmids (10 µg plasmids/100 µL) was dropped onto 1 mg of the CGMs and incubated at 37 °C for 1 h. To prepare empty CGMs, PBS that did not include plasmid was used. Since the volume of the plasmid solution was lower than that theoretically incorporated into microspheres, all the plasmids were completely incorporated into CGMs using this impregnation procedure.

### 4.4. Verification of Targeted Gene Expression in Macrophages

Macrophages were cultured with plasmids containing CGMs for 7 days. We then confirmed that macrophages phagocytosed CGMs using light microscopy and GFP expression in macrophages using fluorescence microscopy (LSM800, Zeiss, Jena, Germany). In addition, we performed an ELISA for HGF (R&D Systems, Inc., Minneapolis, MN, USA) in culture medium. Briefly, 1.0 × 10^6^ macrophages were seeded in a 10 cm plate, and we collected the culture supernatant to confirm HGF expression at 7, 14, 21, and 28 days. Data are expressed as mean ± standard deviation (SD). Differences among groups were examined for statistical significance using one-factor analysis of variance (ANOVA) (Bonferroni/Dunn test). A *p* value < 0.05 was considered statistically significant.

### 4.5. Animals

Eight-week-old specific pathogen-free C57BL6/6J male mice weighing 20–25 g were used in this study (Japan SLC Inc., Shizuoka, Japan). They were housed in standard rodent cages kept at a constant ambient temperature (22 ± 1 °C) and humidity (85%) in the Biomedical Research Centre, Center for Frontier Life Sciences, Nagasaki University, and were exposed to 10 h of light each day. The animals had free access to pelleted rodent food and drinking water. The experimental protocol was approved by the Animal Care and Use Committee and the president of Nagasaki University School (Approval number: 1003301048).

### 4.6. In Vivo Experimental Protocol

**Figure 5:**
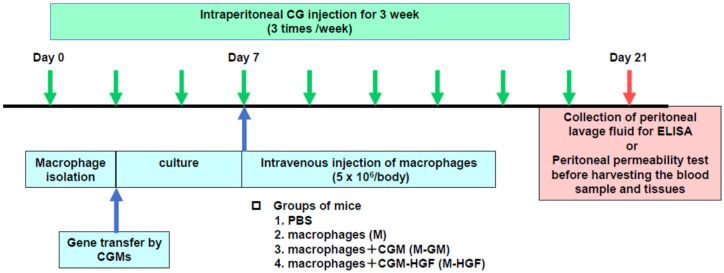
In vivo experimental protocol including timeline.

Peritoneal fibrosis was induced as previously reported [47]. Briefly, under ether anesthesia, mice were injected into the peritoneal cavity with 0.05% CG in 15% ethanol dissolved in 0.2 mL of saline or solvent not including CG 3 times a week for 3 weeks. We obtained macrophages from the peritoneal cavity of mice stimulated with 3% thioglycolate, as described above. Macrophages (5 × 10^6^ cells/body) were cultured with each plasmid (50 µg) and CGMs (1 mg) complex for 5 days under medium containing RPMI 1640 supplemented with 10% FCS, 100 µg/mL penicillin, and 50 µg/mL streptomycin. Macrophages incorporating the plasmid and CGMs complexes were injected intravenously via the tail vein 7 days after the first CG injection. Mice were divided into five groups: (i) mice injected with 15% ethanol dissolved in saline instead of CG intraperitoneally and received PBS instead of macrophages intravenously, defined as the control group; (ii) mice injected with CG intraperitoneally and received PBS intravenously, defined as the PBS group; (iii) mice injected with CG intraperitoneally and received macrophages only intravenously, defined as the M group; (iv) mice injected with CG intraperitoneally and received macrophages incorporating CGMs intravenously, defined as the M-GM group; (v) mice injected with CG intraperitoneally and received macrophages incorporating HGF expressing vector and CGMs intravenously, defined as the M-HGF group. Furthermore, we formed a group of mice injected with CG intraperitoneally and received macrophages incorporating a GFP-expressing vector and CGMs intravenously to confirm the migration of injected macrophages into the peritoneum. Each group comprised 10 mice. Mice had their body weight checked and were sacrificed 21 days after the first CG injection to harvest the blood samples and tissues. Peritoneal tissues were carefully dissected, and the harvested tissues were fixed with 4% paraformaldehyde in PBS (pH 7.4) immediately and embedded in paraffin. Blood samples were obtained by cardiac puncture. Serum aspartate aminotransferase (AST), alanine aminotransferase (ALT), blood urea nitrogen (BUN), and creatinine (Cr) levels were measured using an autoanalyser (Hitachi Ltd., Tokyo, Japan). Figure 5 shows the in vivo experimental protocol including timeline.

### 4.7. Histological and Immunohistochemical Examination

For morphological examination, paraffin-embedded tissues were sliced to 4-µm-thick sections and were stained with hematoxylin and eosin (HE), or immunohistochemical method. For antigen retrieval, deparaffinized tissue sections were treated with proteinase K (P2308; Sigma-Aldrich, Burlington, MA, USA) for 15 min at 37 °C for type III collagen, TGF-β, α-SMA, or autoclaved for 10 min at 121 °C in 10 mM citrate buffer (pH 6.0) for Ki67. To inactivate endogenous peroxidase activity, the sections were treated with 0.3% H_2_O_2_ in methanol for 20 min and then incubated for 30 min at RT with blocking buffer containing 20% normal swine serum, 5% bovine serum albumin, 5% normal goat serum, and 5% FCS in PBS. To detect α-SMA as a marker for myofibroblasts, the sections were incubated for 1 h at RT with a mouse monoclonal α-SMA EPOS antibody (U7033; Dako Japan, Tokyo, Japan). To detect type III collagen, TGF-β, or Ki67, the sections were incubated for 1 h at RT with the primary polyclonal type III collagen, TGF-β, or Ki67 antibodies, which were diluted in the same incubation buffer: rabbit polyclonal type III collagen antibody diluted to 1/400 (LB-1393; LSL Co., Tokyo, Japan), rabbit polyclonal TGF-β antibody diluted to 1/200 (sc-146; Santa Cruz Biotechnology, Inc., Santa Cruz, CA, USA), or rabbit polyclonal Ki67 antibody diluted to 1/500 (Ab15580; Abcam, Cambridge, UK). After reacting with the primary antibodies, the sections were incubated for 30 min at RT with the secondary antibodies diluted in the same incubation buffer: Horseradish peroxidase (HRP)-conjugated swine anti-rabbit antibody diluted to 1/100 (P399; Dako) and either HRP-conjugated rabbit anti-rat antibody diluted to 1/100 (P450; Dako) for type III collagen, biotinylated anti-rabbit IgG antibody and Vectastain Elite ABC Reagent in Vectastain Elite ABC KIT (Vector Laboratories, Burlingame, CA, USA) for TGF-β, or Envision Detection System HRP Labelled Polymer Anti Rabbit (K4003; Dako) for Ki67. By color development using the reaction with H_2_O_2_ and 3,3′-diaminobenzidine tetrahydrochloride, the HRP sites were visualized, and then the sections were counterstained with methyl green and finally mounted. For all the specimens, negative controls were prepared with an irrelevant mouse monoclonal antibody, rat monoclonal antibody, or normal rabbit IgG instead of the primary antibody.

### 4.8. Collection of Peritoneal Lavage Fluid for HGF ELISA

Before sacrifice, peritoneal lavage fluid was collected from five mice in each group. Briefly, mice were infused with 6 mL of PBS into the abdominal cavity. After lightly rubbing the abdomen, the lavage fluid was collected and measured for HGF using ELISA (R&D Systems, Inc., Minneapolis, MN, USA).

### 4.9. Evaluation of Peritoneal Permeability

Before sacrifice, five mice from each group had their peritoneal permeability tested using a simplified PET [32]. Briefly, mice were anesthetized with 0.2 mL of 0.5% Nembutal and infused into the abdominal cavity with 6 mL of peritoneal dialysis solution containing 2.5% glucose (Dianeal, Baxter Healthcare Corporation, Deerfield, IL, USA). All dialysate samples were collected 60 min after infusion to measure the volume and creatinine in the drainage fluid. Immediately after collecting dialysate samples, blood samples were obtained by cardiac puncture. Serum and dialysate creatinine levels were measured using an autoanalyzer (Hitachi Ltd., Tokyo, Japan). The dialysate-to-serum (D/S) ratio of creatinine was used as a parameter of peritoneal permeability.

### 4.10. Data Processing and Statistical Analysis

To assess the peritoneal thickening, we used digitized images by image analysis software (WinROOFver5.5.0, Mitani Corp, Chiba, Japan). In the cross-sections of the abdominal wall, we measured the thickness of the submesothelial compact zone above the abdominal muscle. The image was transformed into a matrix of 1280 × 1000 pixels and viewed at 200× magnification. From the field of view under the microscope, we measured the area of the submesothelial layer at a selected width of 840 µm. Eight areas were selected for each sample, and the average area of the submesothelial layer in HE and type III collagen-positive areas was determined. In addition, within the submesothelial compact zone of each peritoneal sample, the number of α-SMA-, TGF-β-, and Ki67-positive cells was counted in 10 fields at 200× magnification.

Differences among groups were examined for statistical significance using one-factor ANOVA (Bonferroni/Dunn test). Data are expressed as the mean ±SD. Statistical significance was set at *p* < 0.05.

## Figures and Tables

**Table 1:** The body weight and liver and renal functions in each group of mice. Data are expressed as mean ± standard deviation (SD). Phosphate-buffered solution (PBS); CG injected mice administered PBS-CGMs, M; CG injected mice administered macrophages, M-GM; CG injected mice administered macrophages phagocyting CGMs, M-HGF; CG injected mice administered macrophages phagocyting CGMs incorporating *HGF* gene. BW, body weight; AST, aspartate aminotransferase; ALT, alanine aminotransferase, BUN, blood urea nitrogen; Cr, creatinine.

	BW at Day 0 (g)	BW at Day 21 (g)	AST (IU/L)	ALT (IU/L)	BUN (mg/dL)	Cr (mg/dL)
PBS	23.1 ± 1.3	25.8 ± 2.0	54.2 ± 3.8	22.8 ± 2.1	25.6 ± 4.1	0.15 ± 0.01
M	24.0 ± 0.3	25.8 ± 0.2	54.8 ± 1.7	22.5 ± 1.7	24.2 ± 3.3	0.22 ± 0.02
M-GM	24.3 ± 1.0	26.1 ± 1.0	53.0 ± 2.8	23.9 ± 1.8	27.1 ± 1.5	0.21 ± 0.05
M-HGF	25.3 ± 1.0	26.5 ± 0.9	54.0 ± 1.9	24.0 ± 2.4	23.5 ± 3.5	0.20 ± 0.03

## Data Availability

The data presented in the current study are available from the corresponding author upon reasonable request.

## Abbreviations

HGF, hepatocyte growth factor; CGMs, cationized gelatin microspheres; CG, chlorhexidine gluconate; PD, Peritoneal dialysis; siRNA, small interfering RNA; TGF-β, transforming growth factor-beta; GFP, green fluorescent protein; ELISA, enzyme-linked immunosorbent assay; α-SMA, alpha-smooth muscle actin; PET, peritoneal equilibration test; TNBS, 2,4,6-Trinitrobenzene sulfonic acid; EDC, 1-ethyl-3-(3-dimethylaminopropyl) carbodiimide hydrochloride salt; DDW, double-distilled water; RT, room temperature; FCS, fetal calf serum; HE, hematoxylin and eosin; HRP, horseradish peroxidase
